# Desmoplastic melanoma morphology on Thinprep: a report of two cases

**DOI:** 10.1186/1742-6413-4-18

**Published:** 2007-09-19

**Authors:** Becky L Van Ells, James E Madory, Rana S Hoda

**Affiliations:** 1 Department of Pathology and Laboratory Medicine, Medical University of South Carolina, 165 Ashley Avenue Suite 309, Charleston, SC 29425, USA

## Abstract

**Background:**

Desmoplastic melanoma is a variant of malignant melanoma that can range in appearance from sarcomatoid to scar-like. Cytomorphology of desmoplastic melanoma has been previously described on conventional smears; however, to our knowledge, detailed cytomorphology on ThinPrep has so far not been described. Herein, we describe the cytomorphology of two cases of desmoplastic melanoma on fine needle aspiration processed as ThinPrep slides and compare it to that seen on conventional smears. Pertinent immunocytochemical stains, performed on ThinPrep slides are also discussed.

**Case presentation:**

The first case is a woman with a history of desmoplastic melanoma of the scalp with previous local recurrences and lymph node metastasis with a new submandibular mass. The second case is a man with a previously resected desmoplastic melanoma with his first local recurrence. Conventional smears, including air-dried Diff-Quik-stained and alcohol-fixed Papanicolaou-stained smears, demonstrated aggregates of pleomorphic spindle cells admixed with fibrous stroma and single spindle cells. In both cases, nuclei were elongated and plump with irregular nuclear contours, deep grooves, and folds. Chromatin was dark and coarse with either inconspicuous or multiple prominent nucleoli. Cytoplasm was located at the nuclear poles and was fine, wispy, and delicate. The background was clean with no evidence of necrosis or melanin pigment. Papanicolaou-stained ThinPrep slides were prepared from needle rinses and demonstrated excellent correlation of nuclear and cytoplasmic detail of single spindle cells to that seen on conventional smears with the exception of only slight decrease in nuclear size; however, nuclear and cytoplasmic detail of spindle cells embedded in stroma was markedly attenuated. Confirmatory immunostain for *S-100 *protein in both cases was performed on ThinPrep slides demonstrating crisp cytoplasmic staining in the spindle cells.

**Conclusion:**

The cytomorphology of desmoplastic melanoma shows excellent correlation between cytomorphology of single spindle cells on conventional smears and on ThinPrep slides. The major difference noted on ThinPrep slides was attenuated nuclear and cytoplasmic detail of spindle cells embedded in fibrous stoma.

## Background

Desmoplastic melanoma (DM) is an unusual non-pigmented sclerosing variant of malignant melanoma that can range in appearance from sarcomatoid to scar-like. The clinical course often involves many local recurrences and late lymph node metastasis. Herein, we describe the cytomorphology of two cases of DM on fine needle aspiration (FNA) processed as ThinPrep (TP) and compare it to the morphology as seen on conventional smears (CS).

## Case presentation

Case 1 is a 72 year old woman with a history of DM involving the left scalp first diagnosed in November 2004, with multiple local recurrences and parotid lymph node metastasis. The original histologic diagnosis was supported by positive staining for *S-100 *(polyclonal anti-*S-100 *by *DakoCytomation *used in our laboratory shows cells labeled by the antibody to display staining confined to the cytoplasm) and negative staining for *MART-1 *(DAKO), *Desmin *(DAKO), *AE1/AE3 *(DAKO), and *CK903 *(DAKO). In October 2006, she presented with a 3 cm right submandibular mass and underwent a FNA. Case 2 is a 71-year-old man also with a previous history of DM of the left parotid gland diagnosed in November 2005. The original surgical resection specimen showed dense fasicles of pleomorphic spindle cells with nuclear membrane irregularity, prominent nucleoli, and a variable amount of desmoplastic stroma. The immunohistochemical profile was *S-100 *and *Vimentin *positive and *Mart-*1 negative consistent with DM. He underwent total parotidectomy followed by radiation. He presented in January 2007 with a new ill-defined area of "swelling" in his left posterior auricular area at the site of his previous surgery.

In both cases, air-dried Diff-Quik [(DQ) Fisher Scientific] stained smears were made for immediate on-site adequacy and additional smears were fixed in 95% alcohol and stained with Papanicolaou (Pap) stain. The needles were rinsed in Hank's Balanced Salt solution and processed as a Pap-stained TP slide.

**Figure 1:**
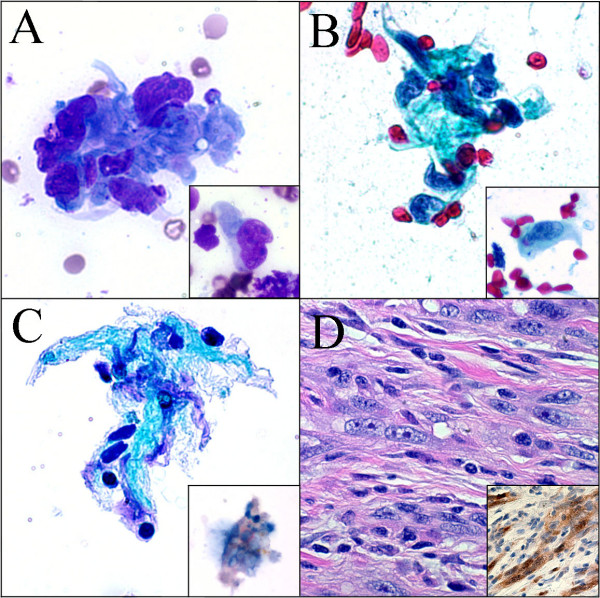
**Case 1**. **A. **Pleomorphic spindle cells embedded in fibrous stroma and occurring singly (*inset*) (DQ 60×). **B. **Similar cells on alcohol fixed smears (Pap 60×). **C. **Cluster of spindle cells in stroma on ThinPrep with some loss of nuclear detail. Cytoplasmic *S-100 *positivity of spindle cells (*inset*) (Pap 60×). **D. **Subsequent surgical resection showing pleomorphic spindle cells in desmoplastic stroma and *S-100 *positivity (*inset*) (HE 40×).

**Figure 2:**
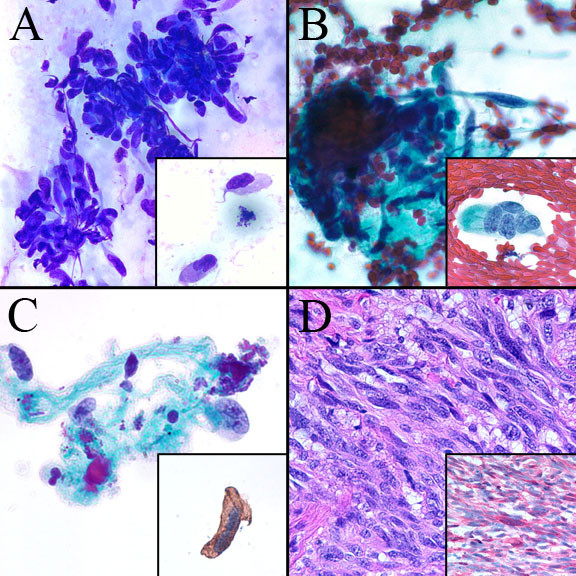
**Case 2**. **A. **Pleomorphic spindle cells in clusters and occurring singly (*inset*) (DQ 60×). **B. **Similar cells on alcohol fixed smears (Pap 60×). **C. **Similar cells on ThinPrep with good nuclear preservation and crisp cytoplasmic *S-100 *positivity (*inset*) (Pap 60×). **D. **Subsequent surgical resection showing pleomorphic spindle cells in desmoplastic stroma and *S-100 *positivity (*inset*) (HE 40×).

The DQ and Pap stained CS were similar in both cases and showed moderate cellularity with pleomorphic spindle cells occurring singly and in small aggregates. The nuclei were plump with deep grooves and folds, chromatin was coarse and clumped, and nucleoli ranged from inconspicuous to multiple and prominent. Intranuclear inclusions were rare. Cytoplasm was small to moderate in amount with a fine wispy character that clung to the nuclear poles. A variable amount of dense stroma was present ranging from small amounts associated with spindle cell aggregates to larger fragments associated with only few spindle cells. The background showed single pleomorphic spindle cells and naked spindled nuclei with no necrosis or melanin pigment (Figure 1A and 1B). Overall, Case 2 CS showed slightly less cellularity, more clusters of spindle cells with less aggregates of stroma, and fewer single cells compared to Case 1 (Figure 2A and 2B).

The TP slides in both cases were made from needle rinses following CS preparation, thus cellularity was low. However, several small aggregates of fibrous stroma with few embedded spindled cells as well as rare single spindle cells similar to those seen in the CS were present. Aggregates of spindle cells seen on CS were not seen on TP. The TP slide for Case 2 showed a slightly increased number of single spindle cells. The single spindle cells showed excellent cell preservation with nuclear and cytoplasmic features identical to that seen on CS. As for cells embedded in stroma, while the nuclei retained their plump character and irregular contour, additional nuclear and cytoplasmic detail was attenuated. The background contained a small amount of blood with no necrosis or melanin pigment, similar to that on CS.

Immunocytochemical staining for *S-100 *was performed on a second TP slide in each case. In Case 1, few spindled cells embedded in stroma showed focal cytoplasmic positivity. This finding combined with the clinical history supported the diagnosis of metastatic DM (Figure 1C). The subsequently excised submandibular mass was diagnosed as consistent with DM with immunohistochemical staining for *S-100 *showing focal positivity similar to that on TP (Figure 1D). In Case 2, single spindle cells showed crisp cytoplasmic *S-*100 positivity. As in Case 1, this finding combined with the clinical history supported the diagnosis of DM (Figure 2C). The subsequent surgical resection of the lesion was diagnosed as DM with positive immunostaining for *S-100 *and *Vimentin *and negative staining for *Mart-1 *(Figure 2D).

## Discussion

Cytomorphology of DM has been previously described in the literature for CS, but has thus far, to our knowledge, not been specifically described for TP. Nance *et *al first described FNA cytomorphology for DM on a patient with a lesion originally diagnosed as lentigo maligna melanoma. The patient underwent a FNA of the second local recurrence in which large tissue fragments of pleomorphic spindle cells and some dissociated spindle cells were described [1]. Chhieng *et al *described a case of DM metastatic to an intraparotid lymph node in which rare fragments of stroma with embedded spindle cells and rare single spindle cells were noted [2]. The nuclear and cytoplasmic features described by these authors are identical to the morphology of our cases described above. Our cases also demonstrate the variable cellularity and the presence of desmoplastic stroma previously described.

Today, liquid based preparation (LBP) is becoming increasingly popular for evaluating non-gynecologic cytology specimens including FNA. A number of morphologic changes are inherent to LBP including altered, reduced or lost background material, smaller and more fragmented cell clusters, smaller cell size, well preserved nuclear detail, more prominent nucleoli, and more easily visualized cytoplasm [3]. Cytomorphology for malignant melanoma on LBP has been previously described. Barkan *et al *[4] compared the CS and TP morphology of melanoma FNA's including epithelioid, spindled, and mixed variants. They described excellent correlation of nuclear and cytoplasmic features, decreased number of intranuclear inclusions, smaller size tissue fragments, and reduction of background material including melanin while intracytoplasmic melanin was retained. Morrison *et al *[5] also described the correlation of melanoma on CS and LBP including epithelioid, spindled, small round, and giant (bizarre) variants. They found an increased detection of melanin and increased cellularity on LBP as compared to CS, with no significant difference in nuclear or cytoplasmic detail or cell shape. While both of these authors describe melanoma variants in their studies, including spindle cell variant, detailed cytomorphology of the spindle cells on CS and TP was not undertaken.

Our morphologic findings on CS correlate with those previously described. The low cellularity of our TP slides was attributed to the slide being prepared from needle rinses of residual material following CS preparation. Nuclear and cytoplasmic features were essentially identical between the two preparation methods with the exception that spindle cells embedded in stroma on TP showed attenuated nuclear and cytoplasmic features. The lack of large tissue fragments and more prominent single cells on TP was likely due to breakup of the tissue during processing of TP as was described by Barkan *et al*.

The differential diagnosis for DM is extensive and includes benign and malignant spindle cell lesions. Some benign lesions in the differential include dermatofibroma, schwannoma, neurofibroma, leiomyoma, and fibromatosis. Malignant lesions include spindle cell carcinoma, dermatofibrosarcoma protuberans, malignant fibrous histocytoma, malignant peripheral nerve sheath tumor, leiomyosarcoma, and fibrosarcoma.

Immunostaining combined with clinical history including age, previous history of DM, and lesion location can be important in distinguishing DM from the above lesions. In both of our cases, cytomorphology and immunohistochemical staining results combined with clinical history was integral in narrowing down our differential diagnoses. Immunostaining on TP can be performed successfully due to the ability to make a second TP slide for immunostaining. This is possible even with scant material, and is especially valuable in cases where cell blo**c**k material is not available [6]. DM is typically positive only for *S-100 *and negative for other melanoma markers including *HMB-45 *and *MART-1 *[7]. In our cases, positive cytoplasmic immunostaining for *S-100 *of both cells embedded in stroma and single cells on TP was helpful in supporting our FNA diagnoses. Our diagnoses were confirmed on follow-up surgical resection of each lesion.

## Conclusion

Our evaluation of two cases of DM cytology on TP showed excellent correlation with CS morphology. Nuclear features and cytoplasmic characteristics of single spindle cells were identical in both CS and TP slides. The only difference noted on TP slides was some loss of nuclear clarity and cytoplasmic detail of spindle cells embedded in fibrous stroma. Confirmatory immunostains can be performed on TP in cases with limited diagnostic material. Awareness of morphologic similarity between CS and TP and the ability to perform immunostains on TP may prove to be of diagnostic utility for cytologists.

## Competing interests

The author(s) declare that they have no competing interests.

## Authors' contributions

BLV carried out background research and drafted the manuscript. JEM participated in image capture and figure design. RSH conceived the idea for the manuscript as well as its design and coordination. All authors read and approved the final manuscript.
